# Self-perception of physical activity and fitness is related to lower psychosomatic health symptoms in adolescents with unhealthy lifestyles

**DOI:** 10.1186/s12889-019-7311-2

**Published:** 2019-07-23

**Authors:** Migle Baceviciene, Rasa Jankauskiene, Arunas Emeljanovas

**Affiliations:** 1 Department of Physical and Social Education, Lithuanian Sports University, Sporto str. 6, LT-44221 Kaunas, Lithuania; 2 Institute of Sport Science and Innovation, Lithuanian Sports University, Sporto str. 6, LT44221, Kaunas, Lithuania

**Keywords:** Adolescents, Psychosocial well-being, Health, Physical activity, Physical fitness

## Abstract

**Abstract:**

The general aim of the present study was to examine how physical activity, participation in sports, and beliefs about personal physical activity and physical fitness are associated with adolescents’ psychosomatic health complaints (PHC) in relation to their lifestyles.

**Methods:**

A total of 3284 11–19-year-old adolescents (average age 14.9 ± 2.0; 48.6% male) participated in the population-based cross-sectional study. Self-administered questionnaires addressed lifestyle, sports participation, physical activity, physical fitness perception, and PHC.

**Results:**

Female gender (OR = 1.92; 95% CI = 1.57–2.35), smoking (OR = 1.31; 95%PI = 1.01–1.68), alcohol consumption (OR = 1.60; 95%PI = 1.30–1.97), unhealthy foods (OR = 1.14; 95%PI = 1.04–1.26), hours of internet use (OR = 1.14; 95%PI = 1.07–1.21), and poor personal fitness perception (OR = 1.60; 95% CI = 1.27–2.02) were associated with PHC in adolescents. Lower physical activity and self-perceived insufficient physical activity, perception of physical fitness as being poor, and not participating in sports were associated with greater somatic and psychological complaints controlling for age, gender, and BMI. Participation in sports and physical activity did not change PHC in adolescents involved in unhealthy behaviour. However, a positive perception of one’s own physical activity and physical fitness decreased PHC in adolescents who reported an unhealthy lifestyle.

**Conclusions:**

Adolescents demonstrating poorer health-related behavioural profiles showed higher PHC. Physical activity and sports participation were related to lower PHC. Positive physical activity and physical fitness perception changed the associations between PHC and unhealthy lifestyle: adolescents perceiving themselves as sufficiently physically active and those evaluating their physical fitness as good showed lower PHC, despite the presence of unhealthy habits (high screen time, drinking alcohol, smoking, and consuming unhealthy foods). It is important to study cognitive factors when exploring the associations between adolescent lifestyles and PHC. These results are important for health promotion and education programmes aimed at improving healthy lifestyle and psychosocial well-being in adolescents.

**Electronic supplementary material:**

The online version of this article (10.1186/s12889-019-7311-2) contains supplementary material, which is available to authorized users.

## Background

Adolescence is a time of rapid psychological, physical, and social change. Consumer culture, the development of technologies, and demanding educational requirements make adolescents in Western countries experience challenges to their subjective well-being and psychosocial distress, which can lead to psychosomatic health complaints (PHC) [1–3]. Psychosomatic symptoms are physical symptoms without definitive organic diagnosis. They develop as a result of psychological reinforcement of physiological needs [4]. There is some evidence that somatic health complaints in combination with psychological health complaints are important components of mental disorders [5]. Further, PHC such as pain, sleep problems, and low mood have been associated with adverse prospective outcomes, such as future psychosomatic problems and development of psychiatric diagnoses [6]. Recent studies show that PHC might be related to a lower chance of entering tertiary education [7].

Prevalence of somatic health complaints, such as back, neck, and shoulder pain, is increasing in adolescents since 1970 [8]. This might be explained by the growing popularity of screen-based activities and sedentary lifestyle, which are related to backache and headache [9]. The majority of studies show that the prevalence of PHC is higher in girls [2, 10–12] and increases with age [2]. The region of Northern Europe has a minor but significant increasing trend in adolescent PHC between 1980 and 2016 [13]. However, PHC in adolescents has not attracted much scientific attention in Lithuania, despite the relatively high overall prevalence of youth psychiatric disorders compared with Western European countries [14]. Therefore, the analysis of the factors associated with Lithuanian adolescents’ psychosocial well-being and lifestyle factors is very important [15]. To the best of our knowledge, this is one of the first studies to address this question in Lithuania.

Lifestyle is an important predictor of adolescents’ PHC. Adolescents demonstrating health-compromising lifestyle behaviours are the most likely to have PHC and vice versa. Studies show that risky behaviours (use of alcohol and smoking), not exercising, being overweight, poor body image, negative self-esteem, poor perception of health, lower sense of coherence, problematic internet use, online gaming for escape reasons, and perceived psychosocial stress are the main predictors of PHC [2, 12, 16–21].

Physical activity plays an important role in the physical and mental health of adolescents. However, the results of the studies exploring the associations of PHC and physical activity are not consistent. Some studies show that physical activity is related to or even plays a protective role for PHC [11, 12, 17, 22–25], while others show no associations [26, 27]. Further, some studies demonstrate positive relationships for boys but not for girls [2, 21]. Physical ctivity of Lithuanian adolescents is insufficient and physical fitness has decreased significantly in the past decades [28]. Therefore, it is important to analyse how physical activity and participation in sports are associated with PHC.

One of the most important markers of adolescent health is physical fitness [29]. It is influenced by genetic factors and physical activity [30, 31]. Studies show that physical fitness is associated with higher quality of life [32], better academic achievement [33], lower tobacco consumption [34], and lower depression [25]. However, there is a lack of studies which explore the role of physical fitness in the development of PHC in adolescents; thus, the present study will fill this gap.

In their prospective study, Lang et al. (2018) demonstrate that cognitive factors are important when analysing the associations between physical activity and mental health [35]. They found that adolescents who believe that their physical activity is sufficient to maintain good health reported better psychological functioning. No differences in actual physical activity were observed between groups. The authors suggested that cognitive factors should be studied more intensively when elucidating the associations between physical activity and psychological functioning in young people. This is important for developing effective new interventions targeting healthy lifestyle and psycho-social outcomes. Thus, it is important to investigate the associations between actual physical activity and PHC, and the perception of physical activity, physical fitness and PHC. To the best of our knowledge, this is one of the first studies to explore this issue.

Improving the wellbeing of adolescents is a major public health problem. Therefore, analysis of predisposing factors is an important scientific issue. There is a lack of studies which analyse adolescent PHC and their complex relationship to lifestyles in terms of alcohol and smoking, nutrition, physical activity, physical fitness perception, and participation in sports. Usually, potential risk factors for PHC are investigated as independent main factors; however, the interaction between those factors may help to explain any magnified or suppressed risk [12]. Physical activity might positively impact changes in important health behaviours; thus, it is important to understand what mechanisms underlie these changes. Given this background, the general aim of the present study was to examine how physical activity, participation in sports, and beliefs about personal physical activity and physical fitness are associated with adolescents’ PHC in relation to their lifestyles.

First, we expected that adolescents who meet physical activity recommendations and participate in sports would demonstrate lower PHC, controlling for age, gender, and BMI. Additionally, based on previous research, we expected that adolescents evaluating their physical activity as sufficient and physical fitness as good would show lower PHC. Our second assumption was that adolescents demonstrating poorer health-related behavioural profiles would show higher PHC. Finally, we expected that physical activity, participation in sports, physical activity evaluation as sufficient, and physical fitness perception as good would be related to lower PHC in adolescents with poorer lifestyle profiles (consuming alcohol and smoking, reporting unhealthy nutrition, and long hours spent sitting).

## Methods

### Study design and study participants

Baseline data was acquired for 3284 11 to 19-year-old schoolchildren (average age 14.9 ± 2.0; 48.6% male) who participated in the population-based cross-sectional study (response rate ~ 90%). Using a nested random sampling method, 20 secondary schools were selected across the country, representing cities, towns, and rural communities. The study included fifth to twelfth grade schoolchildren from secondary schools in the seven major Lithuanian districts. Data entry and analyses were performed at the Lithuanian Sports University. Parents’ written informed consent and children’s assent was obtained prior to data collection. Ethical approval was granted by the Lithuanian Ministry of Education and Science and Kaunas Regional Biomedical Research Ethics Committee (2012-12-14 Nr. BE-2-45).

### Measures

Socio-demographic (age, grade, gender, residence) and lifestyle-related information was assessed using an anonymous questionnaire approved for the national survey of Health Behaviour in School-aged Children (HBSC) study [36] and three additional self-developed questions (participation in sports, self-perceived physical activity and self- perceived physical fitness, Additional file 1).

PHC were assessed using the questions from the HBSC study [36]. Respondents were asked how often they had experienced the following somatic symptoms in the last 6 months: headache, stomach ache, back pain, feeling dizzy, and psychological symptoms – “feeling irritable”, “feeling nervous”, “feeling low” and “difficulties getting to sleep”. Response options for each symptom were (1= “rarely or never”, 2 = “about every month”, 3 = “about every week”, 4 = “more than once a week”, 5 = “about every day”. Next, in the dataset symptoms were summarised to represent scores of somatic and psychological symptoms. Thus, possible scores of psychological (feeling sad, feeling irritable, feeling tense, sleep troubles) and somatic (headache, stomach ache, back pain and feeling dizzy) symptoms ranged from 4 to 20. The higher scores indicate greater symptoms of PHCs. Percentage of at least one PHC about every week and more often was calculated and two groups were created (PHC group and no PHC group).

Physical activity was assessed following the question from the HBSC study [36]. Study participants were asked about the number of days per week that they pursue moderate and vigorous physical activity (MVPA) for at least 60 min a day. The evaluation of physical activity was based on WHO-recommended levels of physical activity (PA) for children [37]. All the students were classified into 2 groups: < 5 days per week (not sufficiently active) and ≥ 5 days per week (sufficiently active).

Participation in sports (PS) was assessed using a single self-developed tool which asked about participation in sports activities (not including school physical education) and the frequency of sessions with answers as following: ‘not participating in sports’, participating in 2–3 times per month’, ‘participating once per week’, ‘participating twice or more times per week’. The responses in relation to participation in sports were used to divide all students into participation in sports (‘2-3 times per month’, ‘participating once per week’ and ‘participating twice or more times per week’) and not participation in sports groups.

Self- perceived physical activity (SSPA) was assessed by question asking to evaluate their own physical activity. The possible answers were: ‘not sufficiently active’, ‘some physically active’, ‘satisfactorily physically active’ and ‘I am very physically active’. Two groups of self-perception of physical activity were formed: the group of self-perceived not sufficient physical activity (‘not sufficiently active’ and ‘some physically active’) and the group of self-perceived sufficient physical activity (‘satisfactorily physically active and ‘very physically active’). This question was self-developed for this study.

Self- perceived physical fitness (SPSF) was assessed by a single question: students were asked to score their own fitness as ‘very fit’, ‘fit enough’, ‘average fitness’, ‘a little unfit’ or ‘very unfit’ when compared with others. The first three categories were combined to represent the group of good physical fitness while the other two were combined to represent poor physical fitness. This question was self-developed for this study.

Screen – time behaviours were assessed using the questions from the HBSC study [36]. Non-educational screen time (TV, computer using the internet, and/or video games) was measured separately for schooldays and weekends as recommended in HBSC study [36]. Students were asked how much time they spend for TV watching, computer games and browsing internet in hours per day. Only screen time of schooldays was analysed in this study. Then, adolescents were divided into two groups: those involved in screen-based behaviour ≤2 h per day and > 2 h per day based on recommendations for children and youth [38].

Nutrition habits were assessed by a food frequency questionnaire approved by the national survey of HBSC [36]. The consumption of foods groups was rated on a 7-point scale ranging from 1 (never) to 7 (every day or several times per day). Unhealthy (sweets, beverages with sugar, fast foods, chips) and healthy (fresh vegetables and fruits) nutrition factors were obtained from principal component analysis summing seven nutrition habits (KMO = 0.70, *p* < 0.0001, 59.5% of total variance explained). Dichotomised variable of unhealthy foods consumption was constructed by dividing unhealthy nutrition factors’ scores using median. Two groups were formed: the group of higher consumption of unhealthy foods and group of lower consumption of unhealthy foods.

Tobacco smoking was assessed using the question from the HBSC study [36]. Children who smoked at least once a week were classified as current smokers.

Alcohol consumption was assessed using the question from the HBSC study [36]. Study participants who reported having been drunk twice or more were classified as risky alcohol consumers.

Body mass index (BMI) was assessed objectively. School nurses measured waist circumference using a standard measure (with an accuracy of 0.5 cm), height (with an accuracy of 0.5 cm), and weight (with an accuracy of 0.5 kg). Adolescents were measured in light clothing without shoes. Children were classified into four body mass categories: thin, normal weight, overweight, and obese using the extended international (IOTF) body mass index cut-offs for thinness, overweight, and obesity [39]. For schoolchildren ≥18 years, adult body mass index standards were used to define underweight (< 18.5), normal weight (18.5–24.9 kg/m2), overweight (25.0–29.9 kg/m2), and obesity (≥30 kg/m2). The results showed that 13.0% of boys and 7.5% of girls were classified as either overweight or obese.

Life satisfaction was estimated using a Cantrils ladder scale from 0 to 10, where a higher score indicated higher life satisfaction. This question is from the HBSC study [36].

### Statistical analysis

Firstly, descriptive statistics and distribution normality testing of continuous variables were performed. In addition, preliminary analyses (χ2, Mann-Whitney U, independent samples t test test) were conducted to examine associations between study variables.

Secondly, multiple logistic regression analysis was performed to reveal the prognostic value of study variables for somatic and psychological symptoms. Two dependent variables were constructed for somatic and psychological complaints: 0 – no complaints; 1 – one or more complaint “about every week”. Unhealthy and healthy nutrition factors were employed as continuous variables in the logistic regression models. The Hosmer–Lemeshow goodness-of-fit test was conducted for all models analysed with logistic regression.

And finally, analysis of covariance was employed to demonstrate somatic and psychological symptom average scores in the groups combined from physical activity (PA), sports participation (SP) self-perception of physical activity (SPPA), self-perception of physical fitness (SPPF), and risky behaviours (screen time, alcohol use, smoking, and consumption of unhealthy foods). Each variable was constructed to represent these subgroups: 1 – physically active (PA) group with or without risky behaviours, 2 – insufficient PA group with or without risky behaviours; 3 – sport participation (SP) group with or without risky behaviours; 4 – not SP group with or without risky behaviours; 5 – self-perceiving themselves as physically active (SPPA) group with or without risky behaviours; 6 – SPPA as not sufficient group with or without risky behaviours; 7 - self-perceiving as fit (SPPF) group with or without risky behaviours; 8 - SPPF as bad group with or without risky behaviours. Paired differences between PA (sufficient/not- sufficient, SP (participate/not participate) and SPPA (sufficient/not sufficient) and SPPF (good/bad) groups with and without risky behaviours were assessed. Post hoc *Bonferroni* test corrections were used for multiple comparisons. Age, gender, and BMI group were employed as the covariates in all models performed. Partial eta squared was calculated to show the estimates of effect size of lifestyle-related behaviours on psychosomatic state.

*P* value < 0.05 was considered to be statistically significant. SPSS version 25.0 was used for statistical analysis (IBM Corp., Armonk, NY, USA).

## Results

The results showed that 64% of all surveyed adolescents reported at least one PHC weekly, 55.3% of them were boys and 72.2% girls. Descriptive analysis revealed clear gender differences in almost all study variables. Boys reported higher smoking, alcohol and unhealthy foods consumption. Further, adolescent boys reported higher physical activity, higher self-perceived physical activity and fitness level as sufficient or good, and higher participation in sports (Table 1). Boys demonstrated significantly higher non - educational screen time (> 2 h per day) as compared to girls. Finally, girls demonstrated higher somatic and psychological complaints and lower life satisfaction.

**Table 1:** Sample characteristics according to gender of 11–19-year-old schoolchildren

Characteristics		Boys *n* = 1588	Girls *n* = 1682	*p*
Age, years, m ± SD		14.8 ± 2.2	15.0 ± 2.2	0.002
Place of residence, %	urban	54.7	61.2	< 0.0001
	rural	45.3	38.8	
Body mass index, %	underweight	7.6	17.5	< 0.0001
	normal	79.5	75.0	
	overweight	11.4	6.1	
	obesity	1.6	1.4	
Higher consumption of unhealthy foods %		56.2	44.4	< 0.0001
Lower consumption of unhealthy foods%		43.8	55.6	
Smoking at least once a week, %		19.3	11.3	< 0.0001
Alcohol intake until feeling drunk ≥2 times, %		39.4	32.3	< 0.0001
Physical activity %	< 5 days/week	56.1	76.2	< 0.0001
	≥5 days/week	43.9	23.8	
Self-perception of physical activity as sufficient, %		70.3	54.1	< 0.0001
Self-perception of physical fitness as good, %		82.5	76.4	< 0.0001
Sport participation, %		53.6	38.6	< 0.0001
Screen time, hours/day, m ± SD		5.0 ± 3.2	4.1 ± 2.7	< 0.0001
Screen time > 2 h/day, %		82.6	70.6	< 0.0001
Psychosomatic health complaints (PHC), %		55.3	72.2	< 0.0001
Somatic complaints score, m ± SD		6.8 ± 3.8	7.9 ± 3.8	< 0.0001
Psychological complaints score, m ± SD		8.2 ± 4.2	9.7 ± 4.3	< 0.0001
Life satisfaction score; m ± SD		7.42 ± 2.42	7.29 ± 2.20	0.001

Note: for categorical variables p value was obtained from chi-square test; age, screen time hours/day and life satisfaction scores were compared by Mann-Whitney U test; psychosomatic health complaints scores were compared by independent samples t test; m – mean, SD – standard deviation, n – number of study participants, *p* – level of statistical significance

Next, we explored the associations between socio-demographic and lifestyle-related factors and somatic state controlling for gender and age (results are not presented in the tables). Female gender (OR = 1.92; 95% CI = 1.57–2.35) and risky behaviours such as smoking (OR = 1.31; 95% CI = 1.01–1.68), alcohol use (OR = 1.59; 95% CI = 1.30–1.95), more frequent consumption of unhealthy foods (OR = 1.14; 95% PI = 1.04–1.26), longer hours using the internet (OR = 1.06; 95% CI = 1.00–1.13), and self-perceived physical fitness and physical activity as poor (OR = 1.53; 95% CI = 1.23–1.90; OR = 1.32; 95% CI = 1.08–1.61) were the strongest predictors of somatic symptoms. Older age was related to less frequent occurrence of somatic complaints. Indeed, every additional score of life satisfaction was associated with a 10% lower likelihood of reporting somatic symptoms. The multiple analysis revealed similar findings in predicting psychological complaints. Female students (OR = 1.84; 95% CI = 1.51–2.26) consuming alcohol (OR = 1.60; 95% CI = 1.30–1.98), unhealthy snacks (OR = 1.10; 95% CI = 1.00–1.21), and adolescents evaluating their physical fitness as poor (OR = 1.60; 95% CI = 1.27–2.02) were more likely to report psychological symptoms such as feeling tense, low, and irritable, and disturbed sleep. Longer hours of daily internet use were associated with greater psychological problems (OR = 1.14; 95% CI = 1.07–1.21). By contrast, every additional score in life satisfaction was related to 15% lower odds of psychological symptoms.

Analysis of covariance revealed that self-perceived insufficient physical activity, perception of physical fitness as bad, and not participating in sports increased the frequency of somatic and psychological complaints controlling for age, gender, and BMI (Table 2). However, lower physical activity was associated with greater psychological complaints, but not somatic complaints.

**Table 2:** PHC mean scores for the groups of physical activity (PA), participation in sports (PS), self-perception of physical activity (SPPA), and self-perception of physical fitness (SPPF) in the sample (m, 95% PI), *n* = 3284^a^

Characteristics		Somatic complaints	F, η^2^, *p*	Psychological complaints	F, η^2^, *p*
PA	< 5 days/week	7.36 (7.19–7.53)	*F* = 0.459 η^2^ = 0.000 *p* = 0.498	9.07 (8.88–9.26)	*F* = 3.862 η^2^ = 0.001 *p* = 0.049
	≥5 days/week	7.26 (7.02–7.50)		8.74 (8.47–9.01)	
SPPA	sufficient	6.95 (6.78–7.12)	*F* = 47.93 η^2^ = 0.016 *p* < 0.0001	8.49 (8.30–8.69)	*F* = 56.13 η^2^ = 0.019 *p* < 0.0001
	insufficient	7.96 (7.73–8.18)		9.71 (9.46–9.96)	
SPPF	fit enough	7.03 (6.88–7.18)	*F* = 67.62 η^2^ = 0.023 *p* < 0.0001	8.59 (8.43–8.76)	*F* = 79.76 η^2^ = 0.027 *p* < 0.0001
	unfit	8.44 (8.14–8.74)		10.30 (9.97–10.64)	
PS	yes	7.15 (6.95–7.35)	*F* = 5.83 η^2^ = 0.002 *p* = 0.016	8.72 (8.50–8.95)	*F* = 7.39 η^2^ = 0.003 *p* = 0.007
	no	7.49 (7.30–7.68)		9.15 (8.94–9.36)	

^a^controlled for gender, age, and body mass index group (analysis of covariance, ANCOVA), *PHC* psychosomatic health complaints, *PA* physical activity, *SPPA* self perceived physical activity, *SPPF* self-perceived physical fitness, *PS* participation in sports, *m* mean, 95% *CI* 95% confidence interval, *F* Fisher’s statistics, η^2^ – partial eta squared, *p* level of statistical significance

Next, we analysed PHC average scores in groups combined for PA, alcohol use, smoking, consumption of unhealthy foods, and screen time (Table 3). Analysis of covariance showed that all models except for the somatic complaints related to screen time were significant. Differences were found when comparing adolescents free from risky behaviours and involved in PA ≥5 days per week and those who demonstrated risky behaviours with more (groups 1 and 3, *p* < 0.05) or less frequent physical activity (groups 1 and 4, p < 0.05). We did not find any paired significant differences in physically active and not physically active groups when comparing adolescents with and without risky behaviours.

**Table 3:** PHC mean scores in the adolescent groups reporting different physical activity (PA) by lifestyle-related habits (m, 95% PI), *n* = 3284^a^

Lifestyle-related habits	Somatic complaints	F, η^2^, *p*	Psychological complaints	F, η^2^, *p*
PA ≥5 days/week, screen time ≤ 2 h/day	6.81 (6.35–7.27)	*F* = 2.17 η^2^ = 0.002 *p* = 0.09	8.04 (7.53–8.56)	*F* = 5.62 η^2^ = 0.006 *p* = 0.001
PA < 5 days/week, screen time ≤ 2 h/day	7.57 (7.20–7.94)		8.76 (8.34–9.17)	
PA active ≥5 days/week, screen time > 2 h/day	7.34 (7.05–7.63)		8.96 (8.64–9.28)	
PA < 5 days/week, screen time > 2 h/day	7.29 (7.10–7.48)		9.18 (8.96–9.39)	
PA ≥5 days/week, non-alcohol-drinking, non-smoking	6.54 (6.23–6.86)	*F* = 27.29 η^2^ = 0.028 *p* < 0.0001	7.88 (7.53–8.23)	*F* = 38.24 η^2^ = 0.039 p < 0.0001
PA < 5 days/week, non-alcohol-drinking, non-smoking	6.85 (6.62–7.07)		8.39 (8.14–8.64)	
PA ≥5 days/week, drinking alcohol and/or smoking	8.31 (7.92–8.69)		10.02 (9.59–10.45)	
PA < 5 days/week, drinking alcohol and/or smoking	8.10 (7.82–8.37)		10.12 (9.82–10.43)	
PA ≥5 days/week, lower unhealthy foods	6.55 (6.21–6.91)	F = 12.44 η^2^ = 0.013 *p* < 0.0001	8.08 (7.69–8.47)	*F* = 13.31 η^2^ = 0.014 *p* < 0.0001
PA < 5 days/week, lower unhealthy foods	7.07 (6.83–7.31)		8.70 (8.43–8.96)	
PA ≥5 days/week, higher unhealthy foods	7.79 (7.46–8.12)		9.25 (8.88–9.63)	
PA < 5 days/week, higher unhealthy foods	7.65 (7.40–7.89)		9.49 (9.21–9.76)	

^a^controlled for gender, age, and body mass index group (analysis of covariance, ANCOVA, *PHC* psychosomatic health complaints, *PA* physically active, *m* mean, 95% *CI* 95% confidence interval, *F* Fisher’s statistics, η^2^ – partial eta squared, *p* level of statistical significance

Further, we compared PHC in the groups of adolescents with different health behaviours and who participated and did not participate in sports controlling for age, gender, and BMI (Table 4). The results show that adolescents participating in sports but involved in risky behaviours did not demonstrate significantly lower levels of somatic and psychological symptoms compared with those adolescents who were not attending sports activities, but also involved in risky behaviours. Thus, sports participation demonstrated no protective role against poorer psychosomatic state in adolescents involved in risky behaviours, with the exception of screen time. We did not find any somatic symptom differences between groups of students who participated in sports and those who had elevated screen time hours per day. However, psychological complaints were less frequent in adolescents who spend more than 2 h per day on screen-based behaviour and involved in sports activities as compared with those not involved in sports activities. The statistical significance in models emerged as an effect of significant differences in PHC between adolescents’ sport participation groups not involved in health compromising lifestyle behaviours (Table 4).

**Table 4:** PHC mean scores in the adolescent groups of participation in sports (PS) by lifestyle-related habits (m, 95% PI), *n* = 3284^a^

Lifestyle-related habits	Somatic complaints	F, η^2^, *p*	Psychological complaints	F, η^2^, *p*
PS, screen time ≤ 2 h/day	7.19 (6.76–7.62)	*F* = 2.21 η^2^ = 0.002 *p* = 0.085	8.42 (7.93–8.91)	*F* = 7.08 η^2^ = 0.008 *p* < 0.0001
No PS, screen time ≤ 2 h/day	7.35 (6.97–7.73)		8.54 (8.11–8.97)	
PS, screen time > 2 h/day	7.11 (6.88–7.34)		8.81 (8.56–9.07)	
No PS, screen time > 2 h/day	7.52 (7.30–7.73)		9.38 (9.14–9.62)*	
PS, non-alcohol-drinking, non-smoking	6.48 (6.21–6.74)	*F* = 29.31 η^2^ = 0.03 *p* < 0.0001	7.88 (7.58–8.18)	*F* = 40.73 η^2^ = 0.04 *p* < 0.0001
No PS, non-alcohol-drinking, non-smoking	6.99 (6.75–7.23)*		8.47 (8.20–8.75)*	
PS, drinking alcohol and/or smoking	8.10 (7.79–8.42)		9.98 (9.63–10.34)	
No PS, drinking alcohol and/or smoking	8.25 (7.94–8.56)		10.21 (9.87–10.56)	
PS, lower unhealthy foods	6.49 (6.20–6.78)	*F* = 15.11 η^2^ = 0.016 *p* < 0.0001	8.06 (7.74–8.39)	*F* = 15.33 η^2^ = 0.016 *p* < 0.0001
No PS, lower unhealthy foods	7.26 (6.99–7.52)*		8.84 (8.55–9.14)*	
PS, higher unhealthy foods	7.65 (7.37–7.93)		9.30 (8.98–9.61)	
No PS, higher unhealthy foods	7.73 (7.46–7.8.01)		9.50 (9.19–9.81)	

*p < 0.05 as compared to participation in sports group; ^a^controlled for gender, age, and body mass index group (analysis of covariance, ANCOVA), *PHC* psychosomatic health complaints, *PS* participation in sports, *m* mean, 95% *CI* 95% confidence interval, *F* Fisher’s statistics, η^2^ – partial eta squared, *p* level of statistical significance

Analysis of the groups of participants with self-perceived physical activity mixed with different health lifestyle behaviours showed the highest PHC scores in the group who evaluated their physical activity as insufficient and who were involved in harmful behaviours, controlling for gender, age, and BMI (Table 5). Positive self-perception of physical activity was associated with lower PHC in adolescents reporting no unhealthy lifestyle and in those, reporting high screen time, consuming unhealthy foods, and smoking and using alcohol.

**Table 5:** PHC mean scores in the adolescent groups of self-perceived physical activity (SPPA) by lifestyle-related habits (m, 95% PI), *n* = 3284^a^

Lifestyle-related habits	Somatic complaints	F, η^2^, *p*	Psychological complaints	F, η^2^, *p*
SPPA as sufficient, screen time ≤ 2 h/day	6.92 (6.57–7.27)	*F* = 17.97 η^2^ = 0.019 *p* < 0.0001	8.12 (7.73–8.52)	*F* = 25.11 η^2^ = 0.026 *p* < 0.0001
SPPA as insufficient, screen time ≤ 2 h/day	7.77 (7.31–8.23)*		9.02 (8.50–9.54)*	
SPPA as sufficient, screen time > 2 h/day	6.90 (6.70–7.10)		8.59 (8.37–8.82)	
SPPA as insufficient, screen time > 2 h/day	8.03 (7.77–8.29)*		9.98 (9.69–10.26)*	
SPPA as sufficient, non-alcohol-drinking, non-smoking	6.39 (6.16–6.62)	*F* = 42.89 η^2^ = 0.043 *p* < 0.0001	7.63 (7.38–7.89)	*F* = 59.70 η^2^ = 0.06 *p* < 0.0001
SPPA as insufficient, non-alcohol-drinking, non-smoking	7.36 (7.07–7.65)*		9.15 (8.83–9.48)*	
SPPA as sufficient, drinking alcohol and/or smoking	7.79 (7.51–8.07)		9.81 (9.50–10.12)	
SPPA as insufficient, drinking alcohol and/or smoking	8.82 (8.47–9.17)*		10.60 (10.21–11.00)*	
SPPA as sufficient, lower unhealthy foods	6.52 (6.27–6.76)	*F* = 24.65 η^2^ = 0.026 *p* < 0.0001	7.97 (7.70–8.25)	*F* = 29.53 η^2^ = 0.031 *p* < 0.0001
SPPA as insufficient, lower unhealthy foods	7.57 (7.26–7.88)*		9.36 (9.01–9.71)*	
SPPA as sufficient, higher unhealthy foods	7.36 (7.12–7.60)		9.00 (8.73–9.28)	
SPPA as insufficient, higher unhealthy foods	8.23 (7.91–8.55)*		10.04 (9.68–10.40)*	

*p < 0.05 as compared to sufficient self-perceived physical activity group; ^a^controlled for gender, age, and body mass index group (analysis of covariance, ANCOVA), *PHC* psychosomatic health complaints, *SPPA* self-perceived physical activity, *m* mean, 95% *CI* 95% confidence interval, *F* Fisher’s statistics, η^2^ – partial eta squared, *p* level of statistical significance

Similar results were found when analysing the role of self-perceived physical fitness (SPPF) in combination with unhealthy behaviours. The results showed that adolescents with worse self-perception about their physical fitness demonstrated the highest scores of PHC (Table 6). Positive physical fitness perception was associated with lower PHC in adolescents without health compromising behaviours and in those reporting high screen time, consuming unhealthy foods, smoking, and using alcohol.

**Table 6:** PHC mean scores in the adolescent groups of self-perceived physical fitness (SPPF) by lifestyle-related habits (m, 95% PI), *n* = 3284^a^

Lifestyle-related habits	Somatic complaints	F, η^2^, *p*	Psychological complaints	F, η^2^, *p*
SPPF as good, screen time ≤ 2 h/day	6.92 (6.61–7.24)	*F* = 23.90 η^2^ = 0.025 *p* < 0.0001	8.15 (7.79–8.50)	*F* = 31.40 η^2^ = 0.033 *p* < 0.0001
SPPF as bad, screen time ≤ 2 h/day	8.56 (7.96–9.16)*		9.73 (9.05–10.40)*	
SPPF as good, screen time > 2 h/day	7.02 (6.85–7.19)		8.72 (8.53–8.92)	
SPPF as bad, screen time > 2 h/day	8.42 (8.07–8.77)*		10.54 (10.15–10.93)*	
SPPF as good, non-alcohol-drinking, non-smoking	6.51 (6.31–6.71)	*F* = 47.8 η^2^ = 0.048 *p* < 0.0001	7.84 (7.62–8.07)	*F* = 64.49 η^2^ = 0.064 *p* < 0.0001
SPPF as bad non-alcohol-drinking, non-smoking	7.72 (7.33–8.11)*		9.62 (9.18–10.06)*	
SPPF as good, drinking alcohol and/or smoking	7.80 (7.55–8.05)		9.74 (9.46–10.02)	
SPPF as bad, drinking alcohol and/or smoking	9.39 (8.92–9.87)*		11.28 (10.75–11.80)*	
SPPF as good, lower unhealthy foods	6.70 (6.49–6.92)	*F* = 29.84 η^2^ = 0.031 *p* < 0.0001	8.13 (7.89–8.37)	*F* = 35.70 η^2^ = 0.037 *p* < 0.0001
SPPF as bad, lower unhealthy foods	7.74 (7.31–8.16)*		9.90 (9.42–10.37)*	
SPPF as good, higher unhealthy foods	7.34 (7.13–7.56)		9.06 (8.82–9.31)	
SPPF as bad, higher unhealthy foods	8.96 (8.53–9.40)*		10.64 (10.15–11.13)*	

*p < 0.05 as compared to good self-perceived fitness group; ^a^controlled for gender, age, and body mass index group (analysis of covariance, ANCOVA), *PHC* psychosomatic health complaints, *SPPF* self-perceived physical fitness, *m* mean, 95% *CI* 95% confidence interval, *F* Fisher’s statistics, η^2^ – partial eta squared, *p* level of statistical significance

## Discussion

The aim of the present study was to examine how physical activity, participation in sports, and beliefs about personal physical activity and physical fitness are associated with adolescents’ PHC in relation to their lifestyles. Based on previous research, we expected adolescents who met physical activity recommendations, participated in sports, and evaluated their physical activity as sufficient and physical fitness as good would show lower PHC. This hypothesis was confirmed, showing that participation in sports, physical activity perception, and perceived physical fitness were associated with lower somatic and psychological PHC in adolescents. However, sufficient physical activity showed no associations with somatic PHC; yet, we observed weak associations between physical activity and psychological complaints. Thus, it can be assumed that physical activity and sports participation are related to lower adolescents’ PHC. The positive influence of physical activity for mental well-being is well documented [23–25]. These findings are in line with other studies [11, 17, 21].

Further, we expected that adolescents demonstrating poorer health-related behavioural profiles would show higher PHC. This assumption was confirmed in our study and adds to the knowledge that adolescents with the greatest psychosomatic symptoms are most likely to engage in unhealthy lifestyles [2, 12, 16–18]. However, the novelty of our finding is that we analysed several health-related habits, such as physical activity, screen – time behaviour, nutrition habits, alcohol consumption and smoking. Our findings show that alcohol consumption, smoking, and self-evaluation of physical activity and physical fitness were most strongly associated with adolescents’ PHC in the present sample, controlling for age, gender and BMI. These findings are in line with other studies [11, 12]. Thus, the present study adds to the knowledge that health education programs combining traditional topics such as development of emotional or social skills might benefit from adding lifestyle – related topics such as physical activity and healthy nutrition, prevention of alcohol and smoking. There is some evidence that this approach might be promising [40].

Further, our study demonstrates that positive evaluation of own physical activity and perception of physical fitness as good are associated with lower PHC in adolescents. These findings coincide with the study of Lang et al. (2018) which demonstrated that adolescent personal beliefs about sufficient physical activity were more closely related to psychological functioning and sleep than the self-reported physical activity [35]. The present study supports these findings. Our study shows that perception of physical activity as sufficient and evaluation of own fitness as good were more strongly associated with lower PHC than physical activity or participation in sports. Other studies demonstrated positive influence of physical fitness on quality of life and lower depression [25, 32, 41]. However, as the present study is of cross-sectional design, it might be that adolescents not having issues with PHC are perceiving themselves as more fit. Therefore, further studies with prospective design might be useful.

Finally, we expected that physical activity, sports participation, self-perception of physical activity and physical fitness would change the associations between PHC and unhealthy lifestyle in adolescents controlling for age, gender, and BMI. In other words, we aimed to test the power of physical activity, participation in sports, and self-perception of physical activity and fitness for PHC of adolescents already leading unhealthy lifestyles (consuming alcohol and smoking, reporting unhealthy nutrition and long screen-time hours). Our study demonstrates that physical activity and participation in sports are positively associated with lower PHC in adolescents with more positive health behaviour profiles; however, the effect of physical activity and sports participation on adolescents with poorer health behaviour profiles was not observed. Nevertheless, the results clearly demonstrate the significant associations between the self-perception of physical activity and physical fitness and PHC in adolescents with unhealthy lifestyles. Those adolescents who perceived themselves as sufficiently physically active and those evaluating their physical fitness as good showed lower PHC, despite the presence of unhealthy habits: higher screen time, drinking alcohol, smoking, and consuming unhealthy foods. Thus, it seems that physical activity and fitness perception, but not physical activity or sport participation per se, are associated with lower PHC in adolescents with poorer healthy behaviour profiles. A possible explanation for these results might be the assumption that physical activity possibly lowers PHC via the mechanisms of elevated self-efficacy, overall self-esteem, optimism or higher self-control. Those variables are associated with increased psychological functioning [21, 25, 26].

However, less than half of the adolescents in the present study met the recommendations for physical activity and, as expected, physical inactivity was higher in girls. Therefore, the meaning of the present results needs to be more deeply analysed; we should avoid the position of stating that perception of physical activity and physical fitness is more important than physical activity and physical fitness itself. The impact of physical activity and physical fitness on adolescents’ health is well documented [23, 24, 38, 42–44]; therefore, we should foster adolescents’ physical activity and participation in sport.

Further, adolescents’ self-reported physical activity might be not accurate. Higher physical fitness perception might be the outcome of previous physical activity in adolescents engaged in low physical activity and sports participation at the moment of research. Self-efficacy in sports might also be developed by families that support physical activity or physical education. Nevertheless, our study shows that it is important to strengthen positive physical self-perception in adolescents, especially in those already reporting negative health-related profiles. Because this is a novel finding, other studies should test these results.

These findings have important implications for the practice of public health, health promotion, and physical education. Firstly, our study adds to the knowledge that physical activity, sports participation and self-perception of physical activity and physical fitness are important variables related to PHC and well-being of adolescents. Further, it is necessary not only to increase physical activity and participation in sports, but to promote positive perception of physical self and trust in one’s own physical abilities. Finally, it is important to educate adolescents to objectively evaluate their physical activity and physical fitness by promoting adequate physical activity and physical fitness evaluation. Increasing positive evaluation of physical abilities might also be promising in health promotion programmes aimed at reducing PHC in adolescents already involved in unhealthy behaviours.

These findings are important for both genders; however, special attention should be payed to the adolescent girls. As expected, we found that somatic and psychological complaints were significantly higher in girls and physical activity in girls was lower. This is in line with other studies [2, 11, 12]. Elevated PHC in girls might be explained by a combination of unhealthy lifestyle choices, such as smoking and not exercising, BMI, body image perception, and self-esteem [45]. Thus, increasing physical self-esteem in health education programmes might help to develop a healthier body image, and to reduce psychosocial stress and the outcomes of unhealthy choices in girls.

### Limitations and strengths

The main limitation of our study is the self-reported data on physical activity and absence of objectively measured physical fitness. Future studies could rely on objectively measured variables. The cross-sectional study design cannot assure the temporality between the cause (risk factor) and health outcomes. Some studies demonstrated that depression in adolescents might decrease their physical activity [46]. Thus, it might be that reduced physical activity poor self-reported physical fitness are the outcomes of psychological complaints. Further, when summarising different sedentary behaviours per day, in some cases they may overlap (i.e., browsing internet and watching TV at the same time). Future studies should address this issue and use optimised questionnaires measuring sedentary behaviours in adolescents.

Among the strengths of this study is the inclusion of nationwide representative data on PHC and lifestyle of adolescents. Another strength of the study is complex analysis of the results controlling for age, BMI and gender. Further, to the best of our knowledge, it is the first study to assess the associations between the physical activity and PHC of adolescents with different health-related behavioural profiles. This study provides useful insights for health promotion programmes aimed at fostering psychosocial health in adolescents.

## Conclusions

Adolescents demonstrating poorer health-related behavioural profiles show higher PHC. Physical activity and sports participation were associated with lower adolescents’ PHC. Evaluation of own physical activity as sufficient and perception of physical fitness as good were associated with lower PHC in adolescents. Positive physical activity and physical fitness perception changed the associations between PHC and unhealthy lifestyle: adolescents perceiving themselves as sufficiently physically active and those evaluating their physical fitness as good showed lower PHC, despite the presence of unhealthy habits (higher screen time, drinking alcohol, smoking, and consuming unhealthy foods). It is important to study cognitive factors when exploring the associations between adolescent lifestyle and PHC. These results are important for health promotion programmes aimed at improving psychosocial well-being in adolescents.

## Additional file

Additional file 1: Additional questions developed for this study. (DOCX 15 kb)

## Data Availability

The datasets generated and/or analysed during the current study are not publicly available but are available from the corresponding author on reasonable request.

## Abbreviations

95% CI: 95% Confidence interval
BMI: Body mass index
OR: Odds ratio
PA: Physical activity
PHC: Psychosomatic health complaints
PS: Participation in sports
SD: Standard deviation
SPPA: Self-perceived physical activity
SPPF: Self-perceived physical fitness
