# Supplementary material for: Self-perception of physical activity and fitness is related to lower psychosomatic health symptoms in adolescents with unhealthy lifestyles
Source: BMC Public Health. 2019 Jul 23;19:980. doi: 10.1186/s12889-019-7311-2 (PMC6647301; doi:10.1186/s12889-019-7311-2)
Supplement: Supplementary file 1 — Additional questions developed for this study. (DOCX 15 kb) [file 12889_2019_7311_MOESM1_ESM.docx]

**Three additional questions developed for the study:**

**Participation in sports (PS)**

*Do you participate in any sports activities (not including school physical education)?*

1. *not participating in sports*
2. *participating 2-3 times per month*
3. *participating once per week*
4. *participating twice or more times per week.*

**Self- perceived physical activity (SSPA)**

*How could you evaluate your own physical activity?*

1. *not sufficiently active*
2. *some physically active*
3. *satisfactorily physically active*
4. *I am very physically active.*

**Self- perceived physical fitness (SPSF)**

*How could you evaluate your own physical fitness when comparing with others?*

1. *very fit*
2. *fit enough*
3. *average fitness*
4. *a little unfit*
5. *very unfit.*
